# Comparative Study of Modified Posterior Operation to Treat Kümmell's Disease

**DOI:** 10.1097/MD.0000000000001595

**Published:** 2015-10-02

**Authors:** Feng Wang, Dachuan Wang, Bingyi Tan, Jun Dong, Rongjie Feng, Zenong Yuan, Naiguo Wang

**Affiliations:** From the Department of Spinal Surgery, Shandong Provincial Hospital affiliated to Shandong University, China.

## Abstract

The present study aimed at examining the curative effect of modified posterior operation on treatment of Kümmell's disease.

About 30 patients of Kümmell's disease with complete image and clinical data treated during June 2004 to July 2013 were conducted with anterior and posterior approaches, respectively. Kyphotic Cobb angle, fractured vertebra wedge angle, and the anterior and posterior heights of fractured vertebra were all measured through x-ray before and after operation, and the pain visual analog scale (VAS) was determined for evaluating the effect of operations. The injury and restoration of neurological function were assessed using Frankel classification.

Patients in group A were treated with anterior operation, whereas group B was posterior operation. Postoperatively, VAS score, kyphotic Cobb angle, anterior vertebra height, and pathologic vertebra wedge angle were all significantly improved in patients with Kümmell's disease receiving modified posterior operation (group B). Similar results were also observed in patients with anterior operation. The improvement of VAS and correction rate of kyphotic Cobb angle indicated equivalent effects of posterior and anterior operations. Meanwhile, alleviated neurological function damage was observed in 2 groups. Relevant factor analysis illustrated that there was no significant correlation of the severity and improvement rate of pain symptoms with age, medical history, anterior and posterior vertebra heights, kyphotic Cobb angle, and pathological vertebra wedge angle.

Compared with traditional anterior approach, modified posterior operation, adopting transpedicular vertebral body grafting combined with vertebral pedicle screw fixation, could produce equivalent effects on kyphosis correction, pain relief, and improvement of neurological function in patients with Kümmell's disease.

## INTRODUCTION

Kümmell's disease is also known as delayed traumatic vertebral collapse disease, and its symptoms mainly include vertebral collapse and kyphosis progression after slight trauma, even accompanied by nerve injury in severe cases.^1^ Currently, patients in mild condition are mainly treated with vertrbroplasty and kyphoplasty. However, due to complicated spinal cord compression, those cases in serious condition have to receive anterior or posterior spin surgery for spina cord decompression, and internal fixation of bone graft was proceeded to reconstruct the spinal stability at the same time.^2,3^ The anterior method commonly needs longer operation time and may result in damage of internal organs.^4^ In addition, it may bring about the prosthesis sinking into osteoporotic spine. Whereas the posterior short-segment fixation is one common treatment for burst fracture,^5–7^ at present, there is yet no consensus on the selection criterion or effects of anterior and posterior approaches.

In this study, we compared the treatment on Kümmell's disease by transpedicular intracorporeal grafting combined with vertebral pedicle screw fixation (modified posterior operation) to the traditional anterior approach and explored appropriate therapy for Kümmell's disease through retrospective comparative analysis.

## DATA AND METHODS

### General Information

A total of 30 patients who suffered from stage III Kümmell's disease were recruited in this study from June 2004 to July 2013. They were all with complete image and clinical data. The study was approved by the ethics committee of Shandong Provincial Hospital Affiliated to Shandong University. Written consents were obtained from all patients. All 30 patients were divided into groups A and B according to surgery. Thirteen cases in group A were all treated with the anterior approach and the other 17 cases in group B all received posterior operation. According to the ASA classification method, the patients were evaluated with the pathogenic condition. Degree I indicates no systematic disease and functional limitation. Degree II indicates mild systematic disease and no functional limitation. Patients with pathogenic condition of degree III show severe systematic disease and certain functional limitation. Patients of degree IV display severe systematic disease and need continuous treatments in entire life. Patients of degree V are dying subjects and more likely to die within 24 h whether surgery is conducted or not. Thirteen cases in group A showed the pathogenic condition of degrees III and IV, of whom 2 were men and 11 were women of age 57 to 79 years with a median age of 68.4 years. Seventeen cases in group B showed similar pathogenic degrees of III and IV. Among patients in group B, there were 3 men and 14 women of age 56 to 79 years with an average age of 69.6 years.

The course of disease in 30 patients ranged from 0.5 to 6 months with an average duration of 3.2 months. Among them, 22 patients had mild trauma history before the onset of illness, including 8 sprains because of lifting weights and 14 cases of flat fall. Two cases suffered from combined rheumatoid and asthma with a long-term use of glucocorticoid. And 1 case undergoing complicated Parkins with long time use of mediation was also included. Common symptoms in patients were intractable chest and low back pains as well as the induction or exacerbation of pains due to position changes. In this study, nerve injury was divided into C grade (13 cases), D grade (14 cases), and E grade (3 cases) according to Frankel classification. All patients’ CT demonstrated the presence of vacuum fractures in vertebral body, and MRI showed that there was limited liquid filling and cavum in vertebral body and that vertebral posterior walls were incomplete and kyphotic. These patients were all single vertebral lesion, 25 of whom were confined to thoracolumber vertebrae (T10-L2). The general data of the patients were detailed in Tables 1 and 2.

**TABLE 1:**
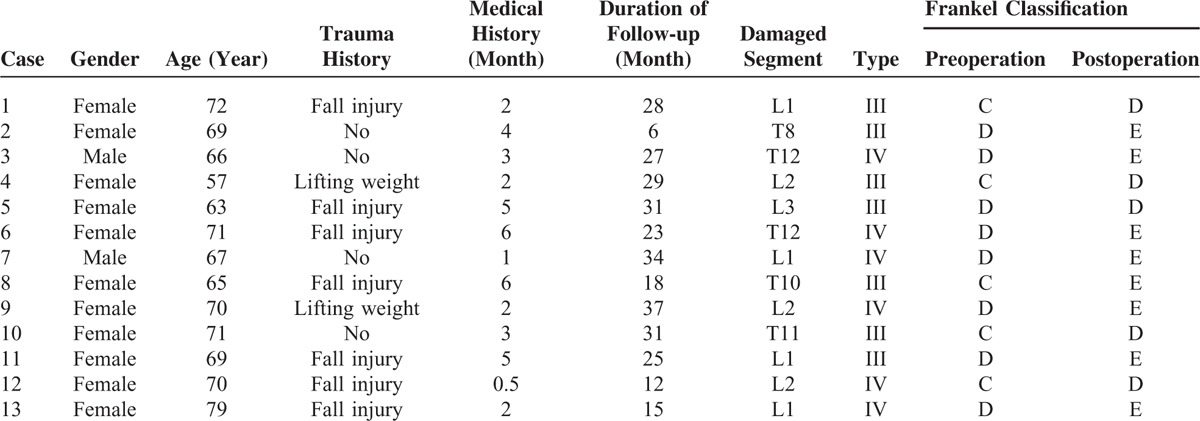
General Data of Patients in Group A

**TABLE 2:**
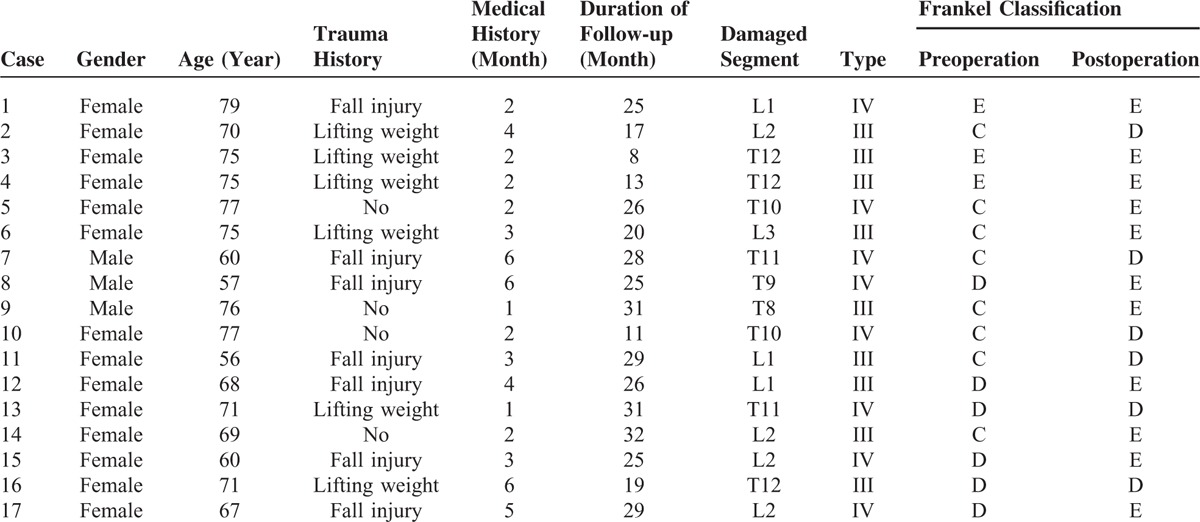
General Data of Patients in Group B

### Operation Methods

All patients received operation under general anesthesia. The requirements in the anterior operation were as follows: adopted right lateral decubitus, shoulder and hip elevated, pathologic vertebra located at the fold of operating table, and fixed patient's spine in a proper extension position. All the above measures were performed to stretch the anterior wall of pathologic vertebra or adjacent intervertebral space to a proper extent, which was beneficial for the intraoperative correction of kyphosis. Pathologic vertebra was resected via left incision through extraperitoneal/thoracic cavity to reduce the decompression of spinal cord. Transvertebra screws were implanted into adjacent vertebrae upper and lower the pathologic vertebra, and then followed by titanium mesh filled with autogous or allogeneic bone after the distraction. Trasvertebra screws were fixed and fused after its reconstruction of spine curve by the prebening titanium rod in accordance with the physiological curvature.

In the posterior operation, patient's chest and ilium were elevated using cushions. Patient was in hyperextension through properly adjusting operating table, and his or her pathologic vertebra was located at the fold of the table. During the operation, the folding operating table was used to prompt the extension of anterior pathologic vertebral wall and assist kyphosis correction. The prone position was adopted in the posterior approach and the incision was seated in the middle of the back. With the channels established transpedicular on both sides of the pathologic vertebra, curet was inserted to the vertebral body to clean granulation tissue and necrotic cancellous tissue until there was only healthy bone tissue on the inner wall. Allograft/autologous bone was implanted in vertebral body and compacted through bilateral pediculates. Another method could also be used, that was to say, the titanium mesh with proper length filled with autologous bone or interbody fusion cage was implanted between the upper and lower bone endplates of pathologic vertebra through the channel established by inserting unilateral pediculate.

### Evaluation Methods

**FIGURE 1:**
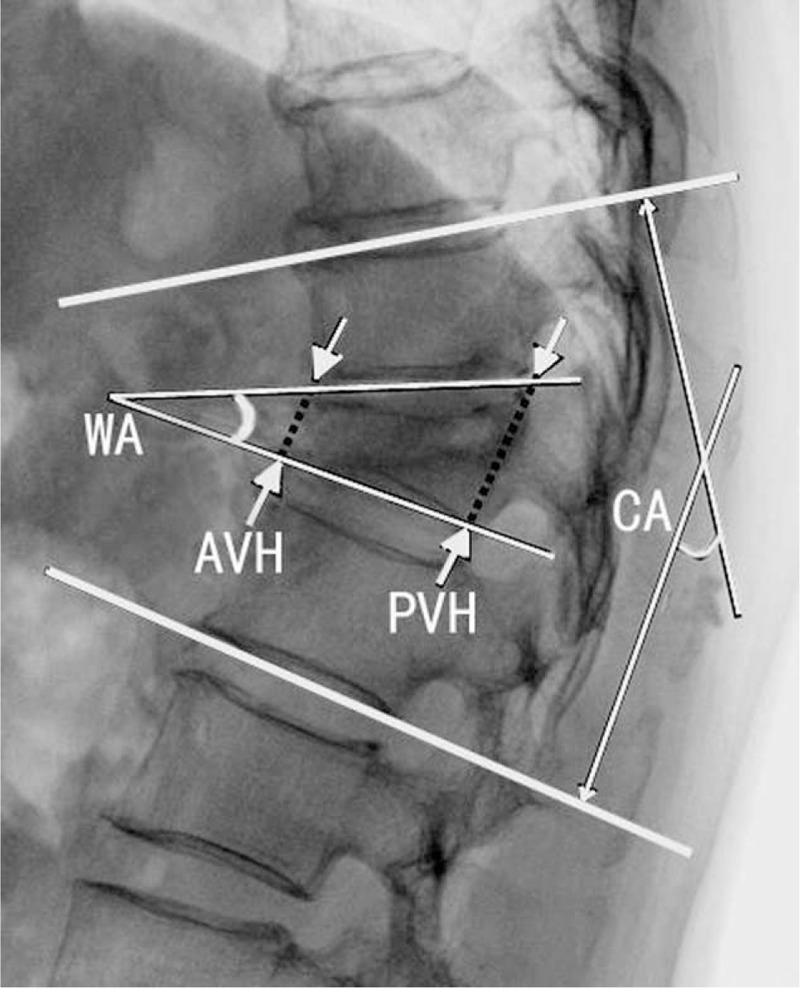
Kyphotic Cobb angle (CA): the angle between the upper and lower endplates of vertebral body with the most serious kyphosis approached up or down by vertebral fracture; wedge angle (WA) of vertebral fracture: the angle between the upper and lower endplates of vertebral fracture; fractured anterior vertebral height (AVH) and fractured posterior vertebral height (PVH): fractured anterior and posterior vertebral heights. AVH = anterior vertebral height, CA = Cobb angle, PVH = posterior vertebral height, WA = wedge angle.

**FIGURE 2:**
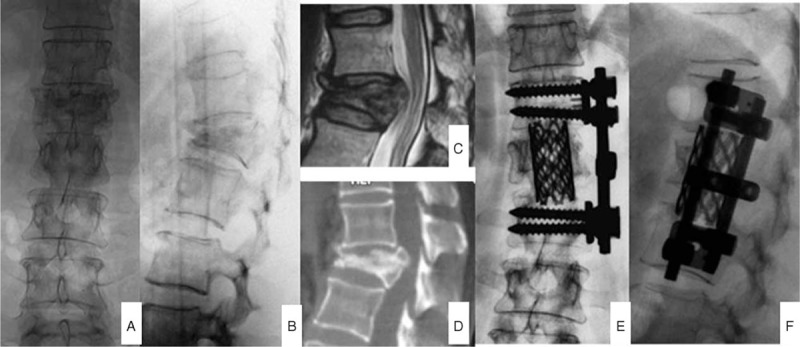
Female patient, 57-year old, was conducted anterior pathologic vertebra resection and internal fixation of titanium mesh supporting grafting bone after 1 month of lower back pain after trauma. Figures A and B represented the anteroposterior and lateral x-ray before operation, showing L1 fracture; figures C and D were MRI and CT before operation, demonstrating L1 fracture and Kümmell's disease; and figures E and F were x-ray after the anterior approach, displaying the fine internal fixation position and satisfactory kyphosis correction.

**FIGURE 3:**
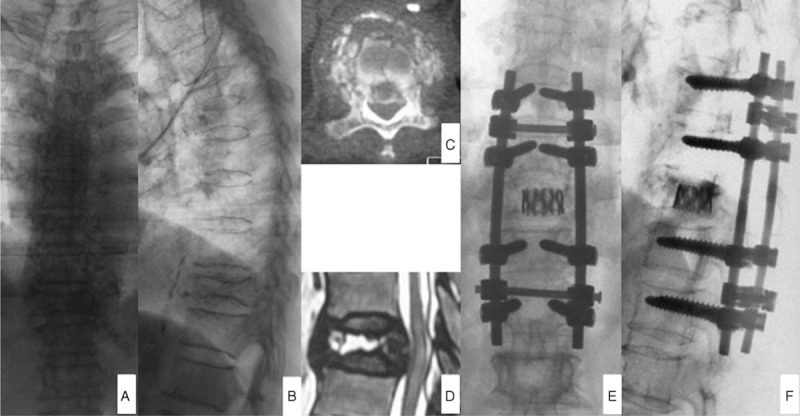
Female patient, 70-year old, was conducted posterior transpedicular internal fixation of titanium mesh supporting grafting bone after 2 months of chest and lower back pain accompanied by lower limbs activity disorder. Figures A and B represented the anteroposterior and lateral x-ray before operation, showing T10 fracture; figures C and D were MRI and CT taken before operation, showing T10 fracture and III stage of Kümmell's disease; and figures E and F were x-ray after anterior operation, manifesting the fine internal fixation position and satisfactory kyphosis correction.

All patients took spinal anteroposterior and lateral x-ray, x-ray in hyperflexion and hyperextension, spine CT and MRI before operation, as well as anteroposterior and lateral x-ray after operation and during the follow-up. Postoperative CT or MRI was taken selectively according to patient's state. Kümmell classification, fracture location, the integrity of posterior vertebral wall, and the compression level on spinal cord were appraised by x-ray, CT, and MRI. Kyphotic Cobb angle, the wedge angle of fractured vertebral body, and the anterior and posterior heights of fractured vertebral body were measured using lateral x-ray (Figures 1–3).^8^ The pain visual analog scale (VAS) was utilized to estimate the relieved degree of pain on patients. The neurological function was evaluated by Frankel classification.

### Statistical Methods

SPSS16.0 statistical software was applied for statistical analysis. All measurement data were conducted with the homogeneity test of variance and the normal test. VAS grades, the anterior and posterior heights of vertebral body, kyphotic Cobb angles, and wedge angles of pathologic vertebra within groups all conformed to homogeneity of variance whether before operation or after operation or during follow-up. All these data were analyzed using the multiple comparative method LSD based on homogeneity of variance of 1-way ANOVA. The following data between groups also accorded with homogeneity of variance, namely VAS grades, VAS improvement rate, the anterior and posterior heights of vertebral body, kyphotic Cobb angle, the correction rate of Cobb angle, and the wedge angle of pathologic vertebra. Several independent samples *t* tests were conducted on comparative analysis between groups. The paired *t* test was performed to compare the differences in every index between 2 time point in each group. Correlation of pain degree and its improvement rate (VAS grade) was evaluated by the linear regression method. α Value of test level was selected on bilateral 0.05.

## RESULTS

### General Results

The operation time of group A was 81.6 ± 21.5 min, obviously longer than that of group B (65.4 ± 17.6 min, *P *< 0.05). The blood loss of patients in group A was 185 ± 52 cc, slightly more than that of group B (178 ± 47cc). The hospitalized time was 5 days (range: 3–12 days) for patients in group A and it was 4 days in (range: 3–10 days) group B. Follow-up was implemented on every patient. Its duration in A group was between 6 and 37 months with a median continuation of 24.3 months (Table 1), whereas in group B the duration ranged from 8 to 32 months with an average of 23.2 months (Table 2).

No patient manifested exacerbation of neurological function damage after operation. Among group A, 8 cases were improved to E grade and 5 cases to D grade (Table 1), whereas 11 cases in group B were elevated to E grade and 6 cases to D grade (Table 2).

By the end of follow-up, there was no such situations as loosening, displacement, and fracture of internal fixation in patients of both groups. In group A, 2 bedridden patients with paralysis developed pulmonary infection and were cured by anti-infection treatment; 1 patient had postoperative transient delirium, but improved after symptomatic treatment. Meanwhile, among patients in group B, 3 developed postoperative transient delirium, 2 had urinary tract infection, and 1 generated pulmonary infection, and they were all cured after conservative treatment.

### Statistical Analysis

Table 3 listed the following data: VAS grades, anterior and posterior vertebral heights, kyphotic Cobb angles, and wedge angle of pathologic vertebra measured before operation, after operation, and at follow-up respectively of patients in 2 groups.

**TABLE 3:**
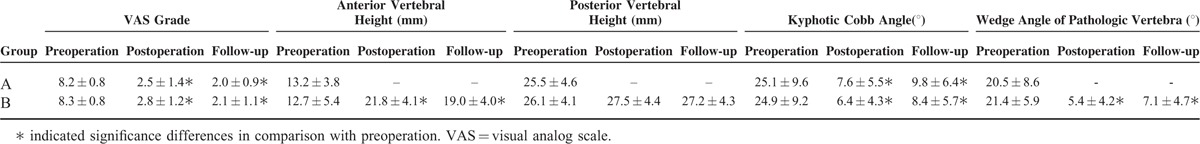
Every Index of Patients in 2 Groups at Preoperation, Postoperation, and Follow-up (n = 30,  ± s)

Data at different time points were analyzed in group A. The differences of VAS grades and kyphotic Cobb angles were all statistically significant between preoperation and postoperation and between preoperation and follow-up (*P* < 0.01; see Table 3). The differences between postoperation and follow-up had no statistical significance (*P* > 0.05; Table 3).

Data of patients in group B at different time points were also analyzed. The results showed that the differences of VAS grades, anterior vertebral heights, wedge angles of pathologic vertebra, and kyphotic Cobb angles had statistical significance between preoperation and postoperation and between preoperation and follow-up (*P* < 0.01; Table 3), whereas the differences between postoperation and follow-up had no statistical significance (*P* > 0.05; Table 3). Posterior vertebral heights among each time point had no statistically significant difference (*P* > 0.05; Table 3).

There was no statistical significance in the differences of VAS grades, anterior and posterior vertebral heights, wedge angles of pathologic vertebra, and kyphotic Cobb angles before operation as well as VAS improvement rate and correction rate of Cobb angle after operation between 2 groups (*P* > 0.05; Table 4).

**TABLE 4:**
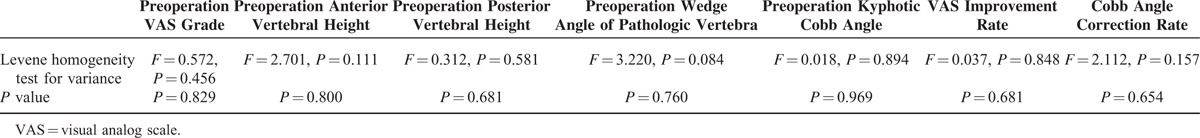
Statistical Comparison of Each Index of Patients Between 2 Groups (*t* Test on Independent Samples)

The association analysis of pain severity showed that the VAS grade before operation had no obvious relationship with age, medical history, anterior and posterior vertebral heights, kyphotic Cobb angle, or wedge angle of pathologic vertebra. Besides, no significant correlation was observed between postoperative VAS grade improvement rate and age, medical history as well as the improvement of anterior and posterior vertebral heights, kyphotic Cobb angle, and pathological vertebral wedge angle (Tables 5 and 6).

**TABLE 5:**
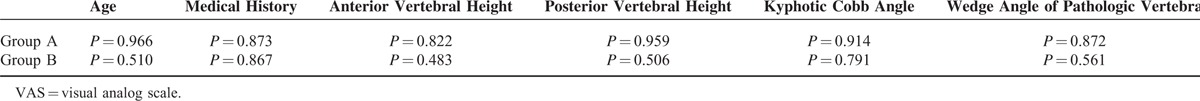
Analysis of Factors Related to VAS Grade Before Operation (Linear Regression)

**TABLE 6:**
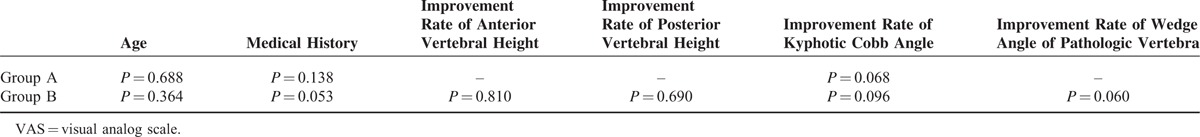
Analysis of Factors Related to the VAS Improvement Rate (Linear Regression)

In a word, these results revealed that posterior operation could produce equivalent effects of anterior operation for Kümmell's disease.

## DISCUSSION

Kümmell's disease is a rare nonunion of osteoporotic vertebral fracture, and its symptoms in clinic include intractable back pain, activity limitation, and neurological dysfunctions.^2,9,10^

Kümmell's disease is divided into 3 stages by Li et al. In stage I, the loss of vertebral height is <20% without degeneration of adjacent segment intervertebral disc; in stage II, the loss of vertebra height is >20%, accompanied by adjacent segment intervertebral disc degeneration frequently, with or without nerve root irritation; and in stage III, posterior vertebral cortex ruptures and dural sac is compressed, coupled with nerve injury or irritation.^11^ At the moment, percutaneous vertebroplasty (PVP) and percutaneous kyphoplasty (PKP) are mainly applied to the patients with mild Kümmell's disease (stages I and II), which are not fit for the patients of stage III.^12–14^ Patients of stage III are mainly treated with spinal grafting fusion, which contains anterior and posterior approaches.^1–3^ As traditional posterior spinal pedicle subtraction osteotomy (PSO) is accompanied by huge trauma, more bleeding and the exacerbation of secondary nerve injury easily caused by shortening of spinal cord, many improved posterior operations have been developed in recent years.^11,15–17^ In the present study, the pathogenic condition of patients were all at stage III according to the above-mentioned method, whereas, according to the ASA classification method, most of patients showed the pathogenic condition of III and IV.

The major cause of using the modified posterior approach is to overcome the shortcoming of huge trauma in traditional posterior osteotomy operation. Compared with the anterior approach and posterior osteotomy operation, the modified posterior operation relatively reduces its surgical trauma and avoids the impact of anterior approach on abdominal viscera function. Moreover, the modified posterior operation can also reach the objectives of the previous 2 surgeries: the restoration of anterior column support, sufficient bone grafting, and the promotion of vertebral bone healing.

The improved posterior operation can effectively restore anterior vertebral height and correct kyphosis with a correction rate of 71.0%, which equals to that of anterior approach (76.7%). No obvious differences in kyphosis correction capability between 2 surgeries are observed (*P* > 0.05). The modified posterior approach has an admirable correction rate compared with other surgeries reported in literatures.^16–18^ But posterior vertebral height had no significant difference among preoperation, postoperation, and follow-up (*P* > 0.05). Reported in the literature, the surgical complication rate in Kümmell's disease ranged from a negligible level to 70% with a kythosis correction rate of 60 to 80%. Correction effect, clinical effect, and complication rate of modified posterior operation were equal to those reported in literatures.^1,2,16^ Due to the surgical method and x-ray measurement, this study did not conduct statistical comparison of anterior and posterior vertebral heights as well as wedge angles of fractured vertebra between 2 groups after operation.

Correlation analysis showed that pain severity and improvement rate had no distinct relationship with age, medical history, anterior and posterior vertebral heights, kyphotic Cobb angle, and pathologic vertebral wedge angle, indicating that there was no need to excessively pursue kyphosis correction and the restoration of vertebral height merely for symptom improvement.

The main purpose of operation on patients of Kümmell's disease is to relieve spinal cord compression, prevent kyphosis progression, and reduce pain.^1–3^ The neurological function could be remarkably improved by both modified posterior operation and anterior approach, and the Frankel classification of neurological function damage was improved from 13 cases of C grade, 14 cases of D grade, and 3 cases of E grade before operation to 11 cases of D grade and 19 cases of E grade after operation (Tables 1 and 2). VAS grade analysis exhibited that the improved posterior operation could significantly relieve pain, and its effect equaled to that of anterior approach and reported in literatures.^2,9,17–19^

There were certain defects in this study, of course. With limited cases, we did not practice comparison study with other posterior approaches, and the circumstances of long-term complication were not clear due to the insufficient follow-up duration. Therefore, these aspects will be the key targets for observation in our future researches.

In conclusion, the modified posterior operation displayed equivalent effects with anterior operation in Kümmell's disease.

## References

[1] Ma R Chow R Shen FH Kummell's disease: delayed post-traumatic osteonecrosis of the vertebral body. *Eur Spine J* 2010; 19:1065–1070. 19949820 10.1007/s00586-009-1205-4 PMC2900014

[2] Li H Liang CZ Chen QX Kummell's disease, an uncommon and complicated spinal disorder: a review. *J Intern Med Res* 2012; 40:406–414. 10.1177/147323001204000202 22613401

[3] Wu AM Chi YL Ni WF Vertebral compression fracture with intravertebral vacuum cleft sign: pathogenesis, image, and surgical intervention. *Asian Spine J* 2013; 7:148–155. 23741556 10.4184/asj.2013.7.2.148 PMC3669703

[4] Kaneda K Asano S Hashimoto T The treatment of osteoporotic-posttraumatic vertebral collapse using the Kaneda device and a bioactive ceramic vertebral prosthesis. *Spine* 1992; 17 (8 Suppl):S295–303. 1523516 10.1097/00007632-199208001-00015

[5] Aebi M Etter C Kehl T The internal skeletal fixation system. A new treatment of thoracolumbar fractures and other spinal disorders. *Clin Orthop* 1988; 227:30–43. 3338219

[6] Kramer DL Rodgers WB Mansfield FL Transpedicular instrumentation and short-segment fusion of thoracolumbar fractures: a prospective study using a single instrumentation system. *J Orthop Trauma* 1995; 9:499–506. 8592263 10.1097/00005131-199509060-00007

[7] Matsuyama Y Goto M Yoshihara H Vertebral reconstruction with biodegradable calcium phosphate cement in the treatment of osteoporotic vertebral compression fracture using instrumentation. *J Spinal Disord Tech* 2004; 17:291–296. 15280757 10.1097/01.bsd.0000097253.54459.a6

[8] Lee SH Kim ES Eoh W Cement augmented anterior reconstruction with short posterior instrumentation: a less invasive surgical option for Kummell's disease with cord compression. *J Clin Neurosc* 2011; 18:509–514. 10.1016/j.jocn.2010.07.139 21315603

[9] Swartz K Fee D Kummell's disease: a case report and literature review. *Spine* 2008; 33:E152–155. 18317183 10.1097/BRS.0b013e3181657f31

[10] Matzaroglou C Georgiou CS Wilke HJ Kummell's disease: is ischemic necrosis or vertebral “microcracking” the first step in the sequence? *Med Hypotheses* 2013; 80:505. 23332323 10.1016/j.mehy.2012.12.003

[11] Li KC Li AF Hsieh CH Another option to treat Kummell's disease with cord compression. *Eur Spine J* 2007; 16:1479–1487. 16568304 10.1007/s00586-006-0094-z PMC2200731

[12] Ren H Wang J Chen J [Clinical efficacy of unipedicular versus bipedicular percutaneous vertebroplasty for Kummell's disease]. *J South Med Univ* 2014; 34:1370–1374. 25263378

[13] van der Schaaf I Fransen H Percutaneous vertebroplasty as treatment for Kummell's disease. *JBR-BTR* 2009; 92:83–85. 19534241

[14] Yang H Gan M Zou J Kyphoplasty for the treatment of Kummell's disease. *Orthopedics* 2010; 33:479. 20608631 10.3928/01477447-20100526-07

[15] Zhang GQ Gao YZ Zheng J Posterior decompression and short segmental pedicle screw fixation combined with vertebroplasty for Kummell's disease with neurological deficits. *Exp Ther Med* 2013; 5:517–522. 23403724 10.3892/etm.2012.833 PMC3570129

[16] Long HQ Wan Y Zhang X Two kinds of posterior approach for Kvmmell's disease after osteoporotic thoracolumbar fracture. *Chin J Traumatol* 2009; 12:142–147. 19486555

[17] Uchida K Nakajima H Yayama T Vertebroplasty-augmented short-segment posterior fixation of osteoporotic vertebral collapse with neurological deficit in the thoracolumbar spine: comparisons with posterior surgery without vertebroplasty and anterior surgery. *J Neurosurg Spine* 2010; 13:612–621. 21039153 10.3171/2010.5.SPINE09813

[18] Eom KS Kim TY Cannula-induced vertebral reduction during Kyphoplasty in a patient with Kummell's disease. *Korean J Pain* 2012; 25:131–132. 22514785 10.3344/kjp.2012.25.2.131 PMC3324741

[19] Hur W Choi SS Lee M Spontaneous vertebral reduction during the procedure of Kyphoplasty in a patient with Kummell's disease. *Korean J Pain* 2011; 24:231–234. 22220246 10.3344/kjp.2011.24.4.231 PMC3248588

